# Complete Genome Sequence of the Highly Transformable *Pseudomonas stutzeri* Strain 28a24

**DOI:** 10.1128/genomeA.00543-14

**Published:** 2014-06-05

**Authors:** Brian A. Smith, Kevin M. Dougherty, David A. Baltrus

**Affiliations:** School of Plant Sciences, University of Arizona, Tucson, Arizona, USA

## Abstract

Here, we report the complete genome sequence for an isolate of *Pseudomonas stutzeri* that is highly competent for natural transformation. This sequence enables insights into the genetic basis of natural transformation rate variations and provides an additional data point for genomic comparisons across a ubiquitous and highly diverse bacterial species.

## GENOME ANNOUNCEMENT

*Pseudomonas stutzeri* is a ubiquitous soil-dwelling bacterium known for its denitrifying capabilities and high genetic diversity (1). *P. stutzeri* strains can be highly competent for natural transformation under laboratory and environmental conditions, which increases the chance of genomic diversification through horizontal gene transfer (2, 3). Strain 28a24 was originally isolated from soil near the Tel Aviv airport, and it displayed one of the highest levels of competence across sampled strains within the initial report (4). Here, we report the complete genome sequence of a rifampin-resistant isolate, strain 28a24 (referred to as DBL332). The potential for high rates of horizontal gene transfer within this isolate, especially compared with those of other *P. stutzeri* strains, makes it an interesting and valuable tool for understanding the evolution of natural transformation systems.

Genomic DNA was prepared from a population initiated by a single colony and purified as per Baltrus et al. (5). The genome was sequenced using PacBio SMRT reads, as well as 100-bp Illumina paired-end reads. Two SMRT cells yielded 177,524 reads, with an average length of 6,383 bp (for a total of 1,133,083,052 bp). These reads were assembled into one contig of 4,731,360 bp using HGAP software (6). The same genomic DNA was used to generate 4,920,635 paired reads from a partial lane of an Illumina HiSeq run. These reads were overlaid onto the HGAP assembly using Geneious 6.0.5, so that bases and indels with >70% support from the Illumina reads were corrected as necessary. Bases and indels with <70% support from the Illumina reads were left as ambiguous. This chromosome was then annotated using the NCBI PGAAP (7), which yielded 4,286 total genes.

### Nucleotide sequence accession numbers.

This whole-genome shotgun project has been deposited in Genbank under accession no. CP007441. The version described in this paper is the first version, CP007441.1.
